# Management of Immune-Related Adverse Events in Patients With Non-Small Cell Lung Cancer

**DOI:** 10.3389/fonc.2021.720759

**Published:** 2021-09-30

**Authors:** Michael Burke, Sawsan Rashdan

**Affiliations:** Department of Internal Medicine, Division of Hematology and Oncology, University of Texas Southwestern Medical Center, Dallas, TX, United States

**Keywords:** lung cancer, immunotherapy, toxicity, adverse events, steroids, checkpoint inhibitor

## Abstract

With proven efficacy of the use of immunotherapy in almost all stages of NSCLC, immunotherapy toxicity has become a very important topic that requires immediate recognition and management. The diagnosis of toxicities associated with immunotherapy in lung cancer can be very challenging and often requires multidisciplinary effort. This mini review gives an overview of the diagnosis and management of immune-related adverse events that arise from using immunotherapy in NSCLC, as well as the potential biomarkers for its early identification and future directions.

## Introduction

With an estimated 228,820 new cases of lung cancer in 2020 and 135,720 anticipated lung cancer deaths comprising 22% of all cancer deaths in the United States, the burden of non-small cell lung cancer (NSCLC) as the most common type of lung cancer and its treatment has become extraordinary (1). Over the past two decades, the care of NSCLC has been revolutionized by the introduction of cancer immunotherapy. Since the initial publications in the management of progressive metastatic disease in Checkmate-057 (2), Checkmate-017 (3), and Keynote-010 (4), immunotherapy has increasingly dominated the management of NSCLC moving to the first-line setting in metastatic disease (5), then with the use in locally advanced disease after concurrent chemoradiation therapy (6) and now the anticipated involvement in the neoadjuvant space (7) (**Table 1**).

**Table 1:** FDA-approved immunotherapy in lung cancer without target mutation.

Trial	Population	Stage	IO combination	Subgroups	Mechanism	Reported irAE (>1%)
PACIFIC	NSCLC	III	Radiation + *durvalumab*		PD-1	Pneumonitis (4.8%)
IMpower150	NSCLC-non-squamous	IV	Carboplatin + bevacizumab + paclitaxel + *atezolizumab*		PD-L1	Dermatitis 29% Hypothyroid 13% Hyperthyroid 4.1% Pneumonitis 2.8% Colitis 2.3% Hepatitis 2%
CASPIAN	SCLC	ES	Carboplatin + etoposide + *durvalumab*		PD-1	Hypothyroid 9% Hyperthyroid 5% Pneumonitis 3% Hepatitis 3% Dermatitis 2% Colitis 2%
IMpower133	SCLC	ES	Carboplatin + etoposide + *atezolizumab*		PD-L1	Dermatitis 18.7% Hypothyroid 12.6% Hepatitis 7.1% Hyperthyroid 5.6% Pneumonitis 2.0% Colitis 1.5%
Checkmate-227	NSCLC	IV	*Ipilumumab* + *nivolumab* + platinum	PD-L1 >1% (Trend <1%)	CTLA-4 PD-1	Skin 34% Endocrine 23.8% Gastrointestinal 18.2% Hepatic 15.8% Pulmonary 8.3% Renal 4.3% Allergic 4.0%
Keynote-189	NSCLC-non-squamous	IV	Carboplatin + pemetrexed + *pembrolizumab*		PD-1	Hypothyroid 6.3% Pneumonitis 4.5% Hyperthyroid 2.7% Dermatitis 1.8%
Keynote-407	NSCLC-squamous	IV	Carboplatin + Taxol/Abraxane + *pembrolizumab*		PD-1	Hyperthyroid 9.2% Hypothyroid 6.4% Pneumonitis 6.4% Hepatitis 4.6% Colitis 2.8% Allergic 1.8% Dermatitis 1.8%
Keynote-042	NSCLC-non-squamous	IV	*Pembrolizumab*	PD-L1 >1% TPS	PD-1	Hypothyroid 12% Pneumonitis 8% Hyperthyroid 6% Dermatitis 2% Allergic 2% Thyroiditis 2% Hepatitis 1% Colitis 1%
EMPOWER-Lung 1 trial	NSCLC	IV	Cemiplimab-rwlc	PD-L1 >50%	PD-1	Dermatitis: grades 3/4: ≥2% Hyperthyroidism 3% Hypothyroidism 7% Colitis 2% Hepatitis 2% Pneumonitis 3%

ES, Extensive stage; NSCLC, non-small cell lung cancer; SCLC, small cell lung cancer.

Currently approved agents for the management of NSCLC include an ever-growing list of immunomodulatory drugs such as pembrolizumab, atezolizumab, nivolumab, durvalumab, and ipilumumab. Unfortunately, the inevitable afterbirth of this revolution has been the recognition of immune-related adverse events (irAEs) of treatment and the need for management of this novel class of complications. Thankfully, the majority of these irAEs are of minor grade and may be treated symptomatically with continuation of treatment; however, due to the nature of immunotherapy, nearly every organ system may be affected and to lethal ends. As will be discussed in the following review, the incidence and severity of these effects in the management of NSCLC may vary depending on drug class, patient characteristics, combination with radiation therapy, and combination with targeted therapy as well as other immunomodulatory drugs.

## Discussion

### Incidence

#### Pneumonitis

The reported incidence of immune-related adverse events has varied since initial observations depending on the immune modulating agent and the clinical setting. Checkpoint inhibitor pneumonitis (CIP) currently occurs in 3%–5% of all cases; however, that estimate rises to 7%–13% in the setting of NSCLC treatment (8). As demonstrated in Checkmate-012, Checkmate-227, and Checkmate-568 (9–11), this incidence worsens when dual checkpoint inhibitor therapy with anti-cytotoxic T-lymphocyte-associated protein 4 **(**CTLA-4) and programmed death-1 (PD-1)/programmed death ligand-1 (PD-L1) is used (12). Furthermore, the increased incidence in NSCLC patients is attributable to the increased association of risk factors for immune-mediated pneumonitis in NSCLC patients including smoking, age >70 years, prior radiotherapy, prior lung disease (including chronic obstructive pulmonary disease), and exposure to the EGFR inhibitor osimertinib (8).

The interaction of checkpoint inhibitor therapy with osimertinib was first reported in the phase 1b TATTON trial assessing its tolerability in combination with durvalumab (13). The occurrence of clinically significant pneumonitis rose from 2.9% in the single agent arm to 38% in the combination arm resulting in the early termination of the trial. Interestingly, this observation remains to be replicated in the phase III multi-arm CAURAL trial (14). Furthermore, this interaction appears to be beyond concurrent treatment with an observed incidence of CIP with osimertinib administration during the first 3 months following checkpoint inhibitor treatment (15).

A similar interaction has been observed with concurrent or prior radiation treatment in NSCLC. In Keynote-001, 13% of patients treated with pembrolizumab and history of prior radiotherapy exposure were observed to develop radiation recall pneumonitis over only 1% incidence in patients without radiation exposure. Comparable numbers were reported in the phase 2 DETERRED trial of concurrent radiotherapy with atezolizumab with an incidence of grade 2 or higher pneumonitis of 10%. Additional observations suggest an association of checkpoint inhibitor pneumonitis with radiation dose with a nearly 9:1 ratio in patients treated with curative-intent radiotherapy over palliative-intent radiotherapy (16, 17).

#### Colitis

Inflammatory colitis following exposure to checkpoint inhibitor therapy represents another significant threat of morbidity and mortality in the management of NSCLC. A recent meta-analysis has demonstrated an overall incidence of 1.4% of colitis associated with immunomodulatory treatment, 0.89% for severe colitis, and 11.62% incidence of diarrhea in patients with NSCLC (18). Similar to the reports for CIP, the combination of CTLA-4 and anti-PD-1/PD-L1 treatment increased the incidence from 0.89% of grade 3 colitis to 3%–5% for combination therapy (9). Moreover, an increased severity is associated with anti-CTLA-4 therapy in addition to an increased incidence of all extraintestinal manifestations including mouth ulcers, anal fissures, and esophagitis/gastritis (19).

Interestingly, while CIP has been observed with increased frequency in the first-line setting, inflammatory colitis appears to increase in incidence with subsequent lines of treatment (18). Furthermore, unlike CIP, no association has been observed between colitis and patient age, sex, smoking, and history of controlled autoimmune disease.

#### Hepatitis

Inflammatory hepatitis is an uncommon cause of treatment interruption in the management of NSCLC with estimates <1% incidence of grade 3 hepatitis and 1%–3% of all grade hepatitis with the use of anti-PD-1/PD-L1 treatment in NSCLC (20, 21). The incidence has been shown to increase dramatically with combination therapy and/or the presence of liver metastases (10, 22).

#### Dermatitis

Dermatologic toxicities are among the most common immune-related adverse events encountered in daily practice when treating lung cancer with an estimated incidence of 44% following CTLA-4 inhibition and 34% with PD-1/PD-L1 targeting treatment (23). Early data have previously suggested that similarity between tumor antigen and somatic epitopes within the skin and fascia may provide a mechanistic explanation for the occurrence of dermatologic events (24). Manifestation of dermatologic adverse events can vary widely in presentation from pruritus and a mild maculopapular rash to bullous pemphigoid or psoriasis flare and even case reports of fulminant Stevens–Johnson syndrome (25). As such, recent National Comprehensive Cancer Network (NCCN) recommendations include a careful dermatologic exam on all patients with planned immunomodulatory treatment to detect and manage any mild or early grade disease before provocation to flare.

#### Endocrinopathy

In Keynote-001 (26), 21% of patients receiving pembrolizumab for the management of NSCLC experienced thyroid dysfunction requiring eventual supplementation. Subsequent clinical experience with immunotherapy of NSCLC has confirmed an estimated incidence of endocrine irAEs of less than 23% with the overwhelming majority involving the thyroid and rarely exceeding grade 2 (27, 28). Hypophysitis secondary to anti-PD-1/PD-L1 therapy is considered extremely rare and more frequently observed secondary to anti-CTLA-4 therapy (29–31). Interestingly, a recent meta-analysis of 38 randomized clinical trials comprising 7,551 patients who underwent checkpoint inhibitor immunotherapy found a consistent reduction in the incidence of thyroiditis and insulin-deficient diabetes for single-agent anti-PD-1/PD-L1 when compared with anti-CTLA-4 monotherapy (28–30, 32). As observed previously, the incidence of all immune-related endocrinopathies was higher with combination therapy (28, 33). Interestingly, the genetic risk for hypothyroidism was associated with risk of developing thyroid immune-related adverse events in NSCLC (34). Furthermore, the occurrence of gastrointestinal, dermatological, and endocrine irAEs in lung cancer patients has been proven to be a predictor of enhanced immune checkpoint inhibitor efficacy (35).

### Diagnosis, Treatment, and Follow-Up

#### Checkpoint Inhibitor Pneumonitis

Diagnosis of checkpoint inhibitor pneumonitis requires a high index of suspicion given the lack of specificity in the presenting symptoms including dyspnea, chest pain, cough, and fever (36). As such, a broad differential diagnosis exists in lung cancer patients including pneumonia, progression of disease, COPD exacerbation, pulmonary embolism, and radiation recall pneumonitis (37). Accordingly, appropriate workup may vary depending on clinical presentation; however, high-resolution computed topography (CT) scan is often useful and is recommended as one of the initial diagnostic tests performed in this setting. In most cases, a multidisciplinary approach is needed for accurate diagnosis. Pulmonary consultation for bronchoscopy with fungal and mycobacterial studies may be considered (38) (**Table 2**)

**Table 2:** Common irAE treatment algorithm.

Symptoms/grade	Workup	Treatment	Follow-up/monitoring
**Pneumonitis**			
Grade 1: Asymptomatic Involving <25% of the lungs	Labs: BNP, CPK, aldolase, CRP Imaging: CT chest WWO Other: EKG, echocardiography Micro: sputum culture, *Mycoplasma*, *Legionella* If febrile*, consult pulmonary medicine for bronchoscopy and infectious workup including pneumocystis testing.	Hold IO therapy 3–4 weeks. Clinical monitoring every 2–3 days.	If persistent, escalate treatment.
Grade 2: Cough/chest pain Dyspnea on exertion without hypoxia		Start PO prednisone 1–2 mg/kg/day tapered over 4–8 weeks. Start broad spectrum antibiotics per local antibiogram.	If unimproved after 48 h, escalate treatment.
Grades 3–4: Dyspnea at rest with or without hypoxemia Involving >50% of the lungs		Transition prednisone to IV 1–2 mg/kg/day methylprednisolone.	If unimproved at 48 h, start infliximab at 5 mg/kg on days 0, 15, and 43. **Permanent discontinuation of immunotherapy.
**Alternative agents:** **Mycrophenolate mofetil BID** **IVIG** **Tocilizumab**			
**Colitis**			
Grade 1: <4 liquid stools above daily baseline	Labs: CBC, CMP, TFTs, CRP Other: fecal fat Micro: stool culture, ova/parasites, CMV PCR, Cdiff PCR, cryptosporidia *If persistent, consider GI referral for colonoscopy or hematochezia. **If peritoneal signs, low threshold CT abdomen WWO and urgent surgical consultation.	Continue IO with symptomatic treatment of loperamide and fluid repletion.	If persistent for >14 days or worsening escalate treatment.
Grade 2: 4–6 liquid stools above daily baseline or new abdominal pain/hematochezia		Start PO prednisone 0.5–1 mg/kg/day *do not wait for colonoscopy.	Clinical monitoring every 72 h, if worsening escalate treatment.
Grades 3–4: >7 liquid stools above daily baseline, life-threatening		Transition to IV methylprednisolone 1–2 mg/kg/daily. Urgent GI consultation.	If unimproved at 72 h, start infliximab at 5 mg/kg on days 0, 15, and 43. **Permanent discontinuation of immunotherapy.
**Alternative agents:** **Mycophenolate mofetil BID** **Tacrolimus** **Hold escalation of immunosuppression until colonoscopy/sigmoidoscopy is performed.			
**Hepatitis**			
Grade 1: AST and ALT <3x ULN Total bilirubin <1.5x ULN	Labs: anti-ANA/SMA/LKM/SLA/LP, iron panel, quantitative Igs Micro: hepatitis A/B/C, HIV, parvovirus, CMV, HSV Other: Imaging: liver US W Doppler *Consider hepatology consult and imaging-guided biopsy if worsening with initial management.	Continue IO.	Repeat CMP weekly.
Grade 2: AST and/or ALT <5x ULN Total bilirubin <3x ULN		Start prednisone 1 mg/kg/day.	Monitor LFTs with INR and albumin biweekly. Escalate management if worsening.
Grades 3–4:		Transition to IV methylprednisolone 2 mg/kg/day.	If no response or worsening at 48 h, start mycophenolate mofetil 500–1,000 mg BID.
**Alternative agents:** **Second-line agents for refractory disease include tacrolimus and anti-thymocyte globulin.			
**Dermatitis**			
Grade 1: Mild rash involving <10% BSA	Physical examination and history excluding other common causes including viral exanthema and drug rash. *Consider urgent referral to dermatology and punch biopsy if refractory to moderate topical steroids or severe symptoms.	Avoid irritants. Consider mild strength topical corticosteroids and PRN oral antihistamine.	Resume routine monitoring.
Grade 2: Symptomatic involving 10%–30% BSA		Escalation to moderate-/high-intensity topical corticosteroids and/or initiation of PO prednisone 0.5–1 mg/kg daily.	Weekly–biweekly physical exam.
Grade 3: Involving >30% BSA or severe symptoms.		Hold IO for severe symptoms and consider admission or IV methylprednisolone at 1–2 mg/kg/day and urgent dermatology evaluation.	
**Alternative agents:** ***Pregabalin, gabapentin, and aprepitant can be considered for management of refractory pruritus.			

Additional considerations:*With prolonged steroid management, calcium/vitamin D supplementation, pneumocystis prophylaxis, and acid suppression. **Avoid infliximab if evidence of hepatic injury.

Regardless of the workup, CIP remains a diagnosis of exclusion, and a stepwise approach to empiric treatment guided by clinical presentation as defined by the CTCAE grading has gained favor (39). Grade 1 CIP presents asymptomatically involving less than 25% of available lung and discovered on surveillance imaging. Accordingly, for grade 1 CIP, a hold of immunotherapy for 3–4 weeks is recommended, and no steroid therapy is needed. Development of dyspnea without oxygen requirement is consistent with grade 2 CIP, and steroid therapy should be initiated with prednisone 1–2 mg/kg/day and tapered over 4–8 weeks. Severe dyspnea with associated hypoxia and involvement of >50% of lung volume on imaging or persistence of grade 2 symptoms for 48 h despite steroid treatment requires escalation of immunosuppressive treatment most commonly with anti-TNFα therapy of infliximab at 5 mg/kg at 0, 2, and 6 weeks (38, 40).

Data for alternative treatment of grade 3 CIP are limited; however, there are encouraging early data for the use of tocilizumab with a 79% response rate (36, 41). Additional discussion regarding the use of mycophenolate mofetil and pooled intravenous immunoglobulin persists, but supportive data remain elusive.

#### Colitis

Management of gastrointestinal toxicity of checkpoint inhibitor therapy often follows a similar algorithmic approach based on clinical presentation (42). Traditional inflammatory bowel disease markers including C-reactive protein (CRP), calprotectin, and albumin have similarly failed to demonstrate an ability to predict the course of immune colitis from checkpoint inhibitor therapy. Endoscopic and histological assessment in the form of Mayo (43), UCEIS (44), and Nancy scores (42) have shown early promise in predicting the need for aggressive immunosuppression to avoid eventual colectomy.

The differential diagnosis of diarrhea and colitis following initiation of checkpoint inhibitor therapy is largely restricted to inflammatory disease, ischemic colitis, and infectious colitis. Endoscopy and directed biopsy may assist in guided initial therapy; however, consideration of infectious etiologies is crucial and a limited workup including stool ova and parasite assay, *Clostridium difficile* polymerase chain reaction (PCR), stool culture, and cytomegalovirus (CMV) serology should be considered in all patients with moderate to severe diarrhea and colitis.

Early grade 1 diarrhea of <4 stools per day may be treated symptomatically with anti-diarrheal medication and fluid replacement. If diarrhea increases to 4–6 stools per day or persists for more than 14 days, immunomodulatory treatment should be held, oral prednisone started at 0.5–1 mg/kg/day, and referral placed for outpatient colonoscopy. Clinical worsening with diarrhea of more than 7 stools per day and/or severe abdominal pain with evidence of peritonitis necessitates hospitalization for resuscitation, intravenous corticosteroids, and initiation of infliximab. Administration of anti-tumor necrosis factor-α therapy has been a mainstay of grade 3–4 treatment; however, a recent case series of seven patients demonstrated effective treatment by targeting gastrointestinal specific integrin with vedolizumab with an observed response in all patients (45).

#### Hepatitis

A broad differential diagnosis exists for the onset of clinically significant transaminitis following initiation of cancer immunotherapy, including infection, autoimmune hepatitis, and drug-induced liver injury. To that end, an expansive workup should be entertained for CMV, herpes simplex virus (HSV), parvovirus, adenovirus, Epstein–Barr virus (EBV), anti-antinuclear antibody (ANA), anti-smooth muscle antibody (ASMA), anti-liver kidney microsomal type 1 antibody **(**LKM-1), quantitative immunoglobulins, an abdominal ultrasound, and often liver biopsy (46, 47).

As the majority of cases are asymptomatic, early intervention is guided by laboratory findings of transaminitis. Of note, mild transaminitis with either AST or ALT below 3 times upper limit of normal (ULN) or total bilirubin below 1.5 times the ULN may be monitored with continuation of therapy. For grade 2 hepatitis with transaminases below 5 times ULN and total bilirubin below 3 times ULN, therapy is held and transaminases are monitored biweekly until levels return to grade 1 or below. Severe hepatitis with transaminases exceeding prior thresholds or evidence of liver failure requires immediate admission for intravenous corticosteroids of methylprednisolone 0.5–1.0 mg/kg/day and consideration of mycophenolate mofetil 500–1,000 mg Q12H if no improvement is observed within 72 h (46, 48).

Historically, anti-TNFα therapy has been discouraged in severe transaminitis secondary to immunomodulatory treatment with the standard escalation to mycophenolate for steroid refractory disease. Here, again, alternative treatments may be considered in the appropriate clinical context with common options including tacrolimus 0.1–0.15 mg/kg/day or anti-thymocyte globulin 1.5 mg/kg/day with consideration of hepatology consultation (49).

#### Dermatitis

Due to the wide variety in dermatologic presentation, an algorithmic approach should be taken in the majority of cases encountered in clinical practice with involvement of specialty care for additional workup and management (50). A mild rash involving <10% body surface area (BSA) with mild symptoms of burning or pruritus may be managed appropriately with medium- to high-potency topical corticosteroids and symptomatic care of oral anti-histamine treatment. Progression to grade 2 rash involving 10%–30% BSA with symptoms inhibiting instrumental activities of daily living would be a reasonable indication for the addition of systemic corticosteroids with prednisone 0.5–1 mg/kg/day with consideration of checkpoint inhibitor hold. Inpatient care and urgent dermatologic consultation may be considered for rashes involving more than 30% BSA depending on severity of symptoms. Provider discretion in addition to patient discussion is critical as many grade 3 rashes with mild symptoms may be reasonably managed in the outpatient setting.

Special consideration should be given to alternative management of checkpoint inhibitor-induced pruritus with gabapentin, pregabalin, and/or aprepitant in cases refractory to antihistamine treatment (51). Consultation of dermatology and disease-directed care should be strongly considered for all cases of grade 4 adverse events including but not limited to drug rash with eosinophilia and systemic symptoms (DRESS) syndrome, toxic epidermal necrolysis, and Steven–Johnson syndrome ahead of permanent discontinuation of checkpoint inhibitor therapy.

#### Endocrinopathies

With the availability of screening assays for many of the observed immune-related endocrine complications of treatment, many are caught early in disease course. Accordingly, in addition to vital signs, routine screening with a basic metabolic panel, calcium, parathyroid hormone (PTH), thyroid stimulating hormone (TSH), free T4, adrenocorticotropic hormone (ACTH), and/or AM cortisol should be obtained ahead of every cycle for the first 6 months and progressively spaced thereafter (33, 50). Otherwise, a high degree of clinical suspicion should be employed for patients undergoing immunotherapy with new or worsening symptoms including fatigue, headache, confusion, diplopia, nausea, vomiting, weakness, weight gain, constipation, diarrhea, sweating, weight loss, polyuria, polydipsia, paresthesia, muscle cramps, lightheadedness, tachycardia, bradycardia, and hypotension (50). Additional workup and management should be guided appropriately with endocrinology consultation for any patient found to be symptomatic or with a positive screen.

In contrast to many other immune-related adverse events, management of endocrinopathy is focused on hormone repletion rather than escalation of immunosuppression and reversal of disease course. Though rare, recognition and diagnosis of adrenal insufficiency is of critical importance for the prevention of adrenal crisis. In the absence of screening, these patients may present with headache, confusion, fatigue, nausea, vomiting, weight loss, and double vision with additional workup directed to explore primary and secondary adrenal insufficiency. With an elevated ACTH with or without hyponatremia and hyperkalemia indicative of primary adrenal insufficiency, additional workup should include an abdominal CT, plasma renin, and 21-hydroxylase antibody serology while administering empiric treatment. In the setting of a depressed ACTH, a pituitary magnetic resonance imaging (MRI), visual field exam, and laboratory workup including prolactin, follicle-stimulating hormone (FSH), luteinizing hormone (LH), estradiol, and testosterone should be entertained.

Asymptomatic or minimally symptomatic patients can be started on oral hydrocortisone 15–25 mg daily in two to three divided doses or oral fludrocortisone 100 mcg daily. Moderately to severely symptomatic patients should be hospitalized for intravenous glucocorticoids of hydrocortisone with 100 mg bolus upfront and then 50 mg every 6 h in addition to aggressive fluid resuscitation with normal saline and thyroid hormone repletion.

#### Rare

Inflammatory arthritis is an increasingly recognized complication of lung cancer immunotherapy with incidence ranging from 1% to 7% (52–54). When suspected, initial workup begins with physical exam and documentation of all involved inflamed joints in addition to laboratory workup of ANA, rheumatoid factor (RF), cyclic citrullinated peptide (CCP) antibodies, and human leukocyte antigen (HLA)-B27 as well as plain films of the involved joints. For mild cases without interruption of activities of daily living, symptomatic care can be pursued with oral prednisone 10–20 mg daily and non-steroidal anti-inflammatory drugs (NSAIDs) as needed for pain relief for 4–6 weeks of therapy with concurrent serial examinations (52). For more severe or refractory cases, additional workup and management should be pursued in coordination with rheumatology referral with consideration of immunotherapy hold.

The incidence of renal toxicity has been reported at <1% for single-agent therapy; however, it has been reported to be as high as 5% with the combination of CTLA-4 and PD-1/PD-L1 therapy (55). Mild elevations in serum creatinine less than 1.5× baseline can be observed with appropriate outpatient hydration; however, elevation above 1.5× baseline should prompt the hold of immunotherapy in addition to consideration for urgent resuscitation and workup with nephrology consultation depending on the degree of renal insufficiency (55). Immune-related neurologic, ophthalmic, and cardiac toxicities are exceedingly rare with reported incidences often <1% with management best guided by subspecialty consultation (50, 56).

### Prediction Biomarker Research

Unfortunately, to date, there are no clinically useful predictive biomarkers to assess immune-related adverse event development in daily practice (57). The association of germline genetic variation with the risk for developing immune-related adverse events when using checkpoint inhibitors is still unclear. Variants in the major histocompatibility complex (MHC) locus were found to be strongly associated with autoimmune diseases in humans (58). Given the strong influence of these genetic variants on autoimmunity, looking at genome-wide single nucleotide polymorphism (SNP) data that are collected from patients treated with checkpoint inhibitors can help in recognizing variants that are associated with irAE. Furthermore, this can help in developing individual polygenic risk scores that can provide a personalized score that measures the genetic risk for an irAE (59). Several retrospective case series have identified discrete class II HLA alleles correlated with the development of several immune-related adverse events ranging from inflammatory arthritis to diabetes mellitus and adrenal insufficiency to colitis (60–65). While these alleles have been associated with the development of immune-related adverse events, they are not wholly predictive; accordingly, their clinical utility even when available remains uncertain.

Interestingly, early diversification of the circulating CD4+ and CD8+ T-cell repertoires following initiation of anti-CTLA-4 treatment has been associated with the onset of immune-related adverse events (66–69). Again, while hypothesis generating, the practical utility of these data remains elusive and is likely outweighed by the burden of recurrent assessment to an uncertain end. Alternatively, serological markers such as surfactant protein, transforming growth factor β1, tumor necrosis factor-α, interleukin 1β, and interleukin 6 for the prediction of radiation-induced pneumonitis have been studied extensively, and their significance toward immunotherapy-induced pulmonary toxicity remains uncertain (70–72).

### Rechallenge of CPI

One of the most pressing questions facing the management of cancer patients undergoing immunomodulatory treatment is the possibility of rechallenge following the occurrence of an immune-related adverse event. With the majority of immune-related adverse events manifesting as low severity grade 1–2 disease, a recent consensus statement from the Society for Immunotherapy of Cancer asserts that rechallenge is reasonable following resolution of event and completion of planned therapy (73). More controversial is the discussion of rechallenge in patients who have undergone a grade 3 event; here, guidance has been largely left to a personalized risk/benefit discussion between patient and provider.

Previously, in a retrospective study, 482 patients undergoing anti-PD-L1 immunomodulatory therapy and suffering treatment interruption secondary to a grade 2/3 immune-related adverse event were observed for possible recurrence on rechallenge (74). Interestingly, while 26% experienced recurrence of the same adverse event and 23% suffered an entirely new immune-related adverse event, 51% of patients did not suffer a recurrent event. A similar occurrence of subsequent events was observed regardless of grade on initial onset, but it did correlate time of initial onset with those events occurring with 3 months of treatment initial most likely to recur.

Further complicating the discussion of rechallenge is the correlation of immune-related adverse event occurrence with disease response. Despite early data suggesting that the occurrence of irAE was predictive of disease response, subsequent studies failed to confirm the initial observation (75–77). Recent data further suggest that early treatment of immune-related adverse events may improve overall survival of those undergoing immunomodulatory treatment by allowing rechallenge and prolonged disease control (74, 76, 78).

### Pre-Existing Autoimmune Disease

Management of lung cancer patients with an indication for checkpoint inhibitor therapy and a history of pre-existing autoimmune disease is an additional point of ongoing debate (79). As a measure to minimize confounding bias, patients with a known history of active autoimmune disease have been excluded from large randomized control trials of immunomodulatory therapy limiting the availability of high-quality data in this population (57, 80). Available retrospective case series assessing anti-CTLA-4 treatment with pre-existing autoimmune disease has emerged in the melanoma literature with a trend toward increased occurrence and severity of irAE when compared with historical controls (81, 82).

Conversely, limited case series suggest that the risk of irAE occurrence with anti-PD-1/PD-L1 therapy in the setting of pre-existing autoimmune disease is comparable to those patients without known history and without identifiable compromise in efficacy (77, 83–85). While these data are encouraging, not all autoimmune conditions bear the same risk of morbidity and mortality on flare. As such, special consideration must be applied to patients with histories of life-threatening autoimmune diseases involving the neurologic and neuromuscular systems such as myasthenia gravis (57). Moreover, in a recent large retrospective cohort, immunosuppression with 10 mg or more of daily prednisone was associated with statistically significant decreases in response rate, progression-free survival, and overall survival for NSCLC patients on anti-PD-1 therapy (86).

Current summary recommendations from the NCCN suggest careful consideration of checkpoint inhibitor therapy in appropriate patients with well-controlled autoimmune disease requiring low to no immunosuppression in coordination with appropriate subspecialty care.

## Conclusion

It is now clear that we need to understand and deal with the respiratory effects of a range of cancer treatments. Although much has been learned regarding the management of immune-related adverse events since their introduction into the NSCLC population, several outstanding questions remain. The lack of reliable, clinically deployable predictive biomarkers and patient characteristics to predict autoimmune development remains an area of active need. Such an assay would allow for the tailored treatment of every patient maximizing the probability of response while minimizing the occurrence of autoimmune phenomena and, thus, harm of treatment. Additional comparative work regarding the incidence of autoimmune events between immunomodulatory classes might partially address this need with lower barrier to entry. Currently, several active clinical trials are addressing this need investigating the correlation of autoantibody and other serological changes in immunotherapy patients with significant adverse event occurrence (NCT03984318, NCT03868046, NCT03409016).

Moreover, as discussed regarding the management of checkpoint inhibitor colitis, while many of the developed treatment algorithms stratify based on universal CTCAE criteria, this often has little correlation with eventual severity of disease, escalation of treatment, duration of treatment, and interruption of immunomodulatory therapy. Additional investigative collaboration across specialties will be required to address this need possibly by the translation of extant tools for the management of known autoimmune disease. Lastly, while anti-TNFα therapy in the form of infliximab has emerged as a rational and consensus standard of care for many forms of steroid refractory disease, often high-quality data remain lacking. To that end, trials investigating novel agents as well as traditional immunosuppressive therapy are ongoing (NCT04375228, NCT04552704).

Thankfully, the overwhelming majority of immune-related adverse events secondary to checkpoint inhibitor therapy appear to be of minor grade with only brief interruptions in treatment if any. Furthermore, with prompt recognition, an algorithmic approach as outlined here and by prior groups can achieve appropriate disease control.

## Author Contributions

All authors contributed to the article and approved the submitted version. UT Southwestern is aware of the submission and agrees to it. The corresponding author is responsible for submitting a competing financial interest statement on behalf of all authors of the paper.

## Conflict of Interest

The authors declare that the research was conducted in the absence of any commercial or financial relationships that could be construed as a potential conflict of interest.

## Publisher’s Note

All claims expressed in this article are solely those of the authors and do not necessarily represent those of their affiliated organizations, or those of the publisher, the editors and the reviewers. Any product that may be evaluated in this article, or claim that may be made by its manufacturer, is not guaranteed or endorsed by the publisher.
